# Cystatin C, a marker for successful aging and glomerular filtration rate, is not influenced by inflammation

**DOI:** 10.3109/00365513.2010.546879

**Published:** 2011-01-04

**Authors:** Anders Grubb, Jonas Björk, Ulf Nyman, Joanna Pollak, Johan Bengzon, Gustav Östner, Veronica Lindström

**Affiliations:** 1 Department of Clinical Chemistry, Lund University Hospital, Lund, Sweden; 2 Competence Centre for Clinical Research, Lund University Hospital, Lund, Sweden; 3 Department of Radiology, University of Lund, Lasarettet Trelleborg, Trelleborg, Sweden; 4 Department of Neurosurgery, Lund University Hospital, Lund, Sweden; 5 Department of Laboratory Medicine, Nicolaus Copernicus University, Collegium Medicum, Bydgoszcz, Poland

**Keywords:** Cardiovascular disease, kidney function, surgery

## Abstract

**Background:**

The plasma level of cystatin C is a better marker than plasma creatinine for successful aging. It has been assumed that the advantage of cystatin C is not only due to it being a better marker for glomerular filtration rate (GFR) than creatinine, but also because an inflammatory state of a patient induces a raised cystatin C level. However, the observations of an association between cystatin C level and inflammation stem from large cohort studies. The present work concerns the cystatin C levels and degree of inflammation in longitudinal studies of individual subjects without infl ammation, who undergo elective surgery.

**Methods:**

Cystatin C, creatinine, and the inflammatory markers CRP, serum amyloid A (SAA), haptoglobin and orosomucoid were measured in plasma samples from 35 patients the day before elective surgery and subsequently during seven consecutive days

**Results:**

Twenty patients had CRP-levels below 1 mg/L before surgery and low levels of the additional inflammatory markers. Surgery caused marked inflammation with high peak values of CRP and SAA on the second day after the operation. The cystatin C level did not change significantly during the observation period and did not correlate significantly with the level of any of the four inflammatory markers. The creatinine level was significantly reduced on the first postoperative day but reached the preoperative level towards the end of the observation period.

**Conclusion:**

The inflammatory status of a patient does not influence the role of cystatin C as a marker of successful aging, nor of GFR.

## Introduction

Increased levels of cystatin C have been shown to be associated with less successful aging than normal levels of cystatin C and cystatin C has been described to have a better diagnostic performance than creatinine to predict successful aging [1–6]. It is known that a decrease in GFR is associated with less successful aging [7,8] and it has therefore been speculated that the advantage of cystatin C as a marker of successful aging is caused by it being a better marker for GFR than creatinine [1,2,9]. However, studies of large patient cohorts have revealed a statistically significant correlation between cystatin C level and degree of inflammation and it has therefore been suggested that cystatin C is a marker not only of GFR but also of the degree of inflammation, thus explaining its superiority as a marker of successful aging [5,10–13]. But, although significant statistical correlations between levels of different components in cohort studies suggest, they do not demonstrate, causal relationships between the levels of the components. The present study was undertaken to verify or refute a causal relationship between the levels of cystatin C or creatinine and the degree of inflammation. It concerns modification of the inflammatory status of patients and its influence upon their plasma levels of cystatin C and creatinine.

## Materials and methods

### Study population

Thirty-five patients scheduled for elective surgery, with normal body temperatures and able to provide written informed consent, without the need for a proxy respondent, were considered for inclusion in the study. The study complied with the Declaration of Helsinki as revised in 1983. Tissue trauma during surgical procedures varied from minor to medium.

### Determination of protein and creatinine levels

Venous blood samples were collected in EDTA-containing tubes on the day before surgery (Day 0) and daily postoperatively for seven days (Day 1 to 7).

Plasma CRP, orosomucoid (alpha-1-acid glycoprotein) and haptoglobin were determined by immunoturbidimetry using a Roche-Hitachi cobas 6000 analysis platform and with a calibrator based upon the international calibrator CRM 470 [14]. Reference ranges for orosomucoid: 0.5–1.2 g/L, and for haptoglobin: 0.3–2.0 g/L [14]. The median CRP-levels in the third and seventh decades of healthy individuals in the general adult population have been given as 1 and 2 mg/L, respectively [15]. Plasma cystatin C was measured by an automated particle-enhanced immunoturbidimetric assay [16] using the same Roche-Hitachi cobas 6000 analysis platform and a calibrator of isolated non-truncated recombinant human cystatin C [16]. Reference range: 20–50 years of age: 0.70–1.21 mg/L and, above 50 years of age: 0.84– 1.55 mg/L [17]. Plasma SAA was measured by an automated particle-enhanced immunonephelometric assay [18] using a Siemens BN ProSpec analysis system and reagents from Siemens. The SAA-values were calibrated against the 1st international standard 1997 serum amyloid A protein lot 92/680 [19]. Reference range: 0.84–11.4 mg/L [18]. Plasma creatinine was determined by a creatininasebased procedure using the same Roche-Hitachi cobas 6000 analysis platform as described above. The creatinine calibrator was traceable to primary reference material with values assigned by isotope dilution mass spectrometry [20]. Reference ranges for males 20–70 years of age: 56–103 µmol/L and for females 20–70 years of age: 42–82 µmol/L [21].

### Statistical analysis

All statistical procedures were performed using SPSS release 15.0.0 (SPSS Inc., Chicago, US). To limit the problem of multiple comparisons, the time course of the levels of each inflammatory marker (CRP, SAA, haptoglobin and orosomucoid), as well as of creatinine and cystatin C, were evaluated by two distinct statistical tests only: (i) Differences between preoperative levels and the first (CRP, SAA, creatinine and cystatin C) or second (haptoglobin and orosomucoid) postoperative day were tested using Wilcoxon signed rank test; and (ii) for CRP, SAA, creatinine and cystatin C, log-linear trends in the changes in levels on postoperative day 1 through 7 were tested overall, using a linear mixed model with the logarithm of the marker day 1 through 7 as dependent variable, day as a fixed continuous covariate and with no random effect but a first-order autoregressive model for the residuals. To assess the correlation between infl ammatory markers and creatinine and cystatin C, the mixed models with inflammatory markers as dependent variables were extended by including the natural logarithm of creatinine and cystatin C, respectively, as fixed covariates. Due to missing data at several time points, mixed models were preferred to methods requiring complete data at all time points. *p*-values < 0.05 were considered significant.

## Results

The presence of systemic inflammation in 35 patients scheduled for elective surgery was investigated by analysis of the plasma levels of four commonly used markers for inflammation: CRP, SAA, haptoglobin and orosomucoid. The preoperative CRP levels in 20 of the patients were below 1 mg/L, which is below the median CRP level in the third decade of a general adult population [15] indicating absence of systemic inflammation in these 20 patients, which therefore were selected for further study. The preoperative levels of orosomucoid and haptoglobin were below the upper reference levels for all or 18, respectively, of the 20 patients, supporting the absence of pronounced inflammation. However, the preoperative level of SAA was slightly above the upper reference level for 8 of the 20 patients with a maximal level of 22.5 mg/L, indicating that a low-degree infl ammation might have been present in some of the patients. The levels of cystatin C, 0.72–1.45 mg/L, and creatinine, 49–115 µmol/L, were below, or just above, the upper reference limits.

**Figure 1:**
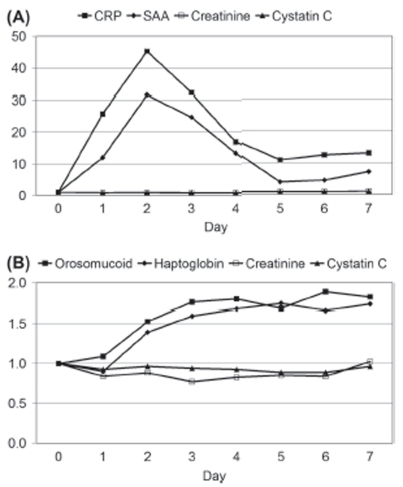
Changes in plasma levels of (A) CRP, SAA, creatinine and cystatin C and of (B) orosomucoid, haptoglobin, creatinine and cystatin C during seven consecutive days after elective surgery. Day zero denotes the day before surgery. The ordinates represent the median level of each analyte in multiples of the preoperative level.

The levels of CRP and SAA increased markedly on the first postoperative day (*p* < 0.001 and *p* < 0.001, respectively) and reached maximal values on the second postoperative day with median levels corresponding to 46 and 32 times the preoperative levels of CRP and SAA, respectively (Figure 1A). Both the SAA and CRP levels decreased after the second day but were still above the upper reference levels on the seventh postoperative day (Figure 1A). The levels of haptoglobin and orosomucoid were significantly increased on the second postoperative day and did not decrease significantly during the following five postoperative days (Figure 1B).

**Figure 2:**
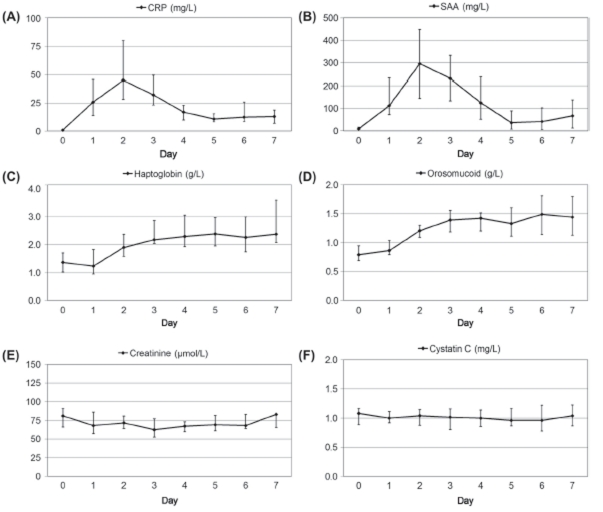
Changes in plasma levels of (A) CRP, (B) SAA, (C) haptoglobin, (D) orosomucoid, (E) creatinine and (F) cystatin C during seven consecutive days after elective surgery. Day zero denotes the day before surgery. The ordinates represent the median and the 1 st and 3 rd quartiles of the level of each analyte.

In contrast to the levels of the infl ammatory markers, the cystatin C level did not change significantly on the first postoperative day (*p* = 0.20; preoperative median = 1.08 mg/L, first postoperative day median = 1.00 mg/L), and remained stable during day 1–7 (*p* = 0.49). Creatinine decreased from 81–65 µmol/L in median on the first postoperative day (*p* = 0.009), but tended to increase again during day 1–7 (*p* = 0.19). The median values and the corresponding 1 st and 3 rd quartiles of the CRP, SAA, haptoglobin, orosomucoid, creatinine and cystatin C levels are given in Figure 2A–F.

The cystatin C level showed no statistically significant correlation with the levels of any of the inflammatory markers (SAA: *p* = 0.10, CRP: *p* = 0.65, Haptoglobin: *p* = 0.72, Orosomucoid: *p* = 0.58). The creatinine level displayed a significant correlation with the SAA level (*p* = 0.019), but not with the levels of any of the other infl ammatory markers (CRP: *p* = 0.13, Haptoglobin: *p* = 0.70, Orosomucoid: *p* = 0.80).

## Discussion

It has been suggested that cystatin C is a better marker than creatinine for survival success not only because it is a better marker for GFR but also because the presence of an inflammatory reaction in an individual produces a raised cystatin C level [5,10–13]. This suggestion has been based upon demonstration of a statistically significant correlation between the levels of CRP, a marker for inflammation, and cystatin C in large cohort studies. However, correlations in cohort studies can suggest, but not demonstrate, causal relationships between the levels of the components. The present investigation comprised testing of the inflammatory status of 20 patients scheduled for elective surgery by studying their plasma levels of four inflammatory markers – CRP, SAA, haptoglobin and orosomucoid before surgery and during seven consecutive postoperative days. CRP and SAA were selected because their plasma levels are known to increase rapidly and extensively after initiation of an inflammatory process and swiftly decrease after disappearance of the cause of the infl ammation. Haptoglobin and orosomucoid were selected because inflammation produces a more sustained increase of their plasma levels than those of CRP and SAA. The 20 patients were selected from a larger cohort of patients scheduled for elective surgery, because their CRP levels were below 1 mg/L, thus indicating the absence of inflammation in these 20 patients. The levels of cystatin C and creatinine in 19 of the 20 patients were below the upper reference limits. As expected, surgery caused a marked increase in the plasma levels of all four markers of infl ammation. In contrast, no change in the cystatin C levels could be observed during the seven postoperative days. Neither did the level of cystatin C show any significant statistical correlation with the levels of any of the four markers of inflammation. We believe that these data strongly argue against the suggestion that systemic inflammation causes increased plasma levels of cystatin C. However, if infl ammatory conditions without alterations in the levels of any of the four inflammatory markers used in this work exist, such conditions might theoretically increase the cystatin C level.

Although the results of the present study might seem to be in confl ict with the previous observations of significant correlations between the plasma levels of CRP and cystatin C in cohort studies [5,10–13], we do not think this is the case. For the cohort studies concerned the risk of cardiovascular events and death in elderly populations and demonstrated that higher levels of both cystatin C and CRP were significantly correlated with higher incidence of cardiovascular events and premature death. This means that even without a causal relationship between the levels of CRP (“inflammation”) and cystatin C, a statistical significant correlation will be found because raised levels of both components indicate increased risk of cardiovascular events and premature death.

The present study indicates that the advantage of cystatin C compared to creatinine as a marker of successful aging is not due to the fact that an increased level of cystatin C signals the presence of infl ammation in addition to a decrease in GFR, as previously suggested [5,10–13]. The advantage might, of course, solely be related to the fact that cystatin C is a better marker for a decrease in GFR than creatinine, but additional causes for its superiority as a marker for successful aging might be suggested. For example, cystatin C might identify the alteration in the composition of the glomerular filtrate with a reduced filtration of large molecules, e.g. cystatin C (13 343 Da), but an unaltered filtration of small molecules e.g. water (18 Da) and creatinine (113 Da), which often occurs before a decrease in GFR can be demonstrated [22–25]. The capacity of cystatin C to early identify an abnormal filtration quality might also explain the observation that an elevated cystatin C level indicates higher incidence of cardiovascular events and death even in the presence of a normal GFR [4,8]. The observations reported in two recent studies of cystatin C in hypertensive patients and in patients on hemodialysis agree with the result of the present study that there is no correlation between cystatin C and CRP in important patient groups [26,27].
